# Proteomic profiling of mitochondria: what does it tell us about the ageing brain?

**DOI:** 10.18632/aging.101131

**Published:** 2016-12-15

**Authors:** Thomas Ingram, Lisa Chakrabarti

**Affiliations:** ^1^ SVMS, Faculty of Medicine, University of Nottingham, Sutton Bonington, LE12 5RD, UK

**Keywords:** mitochondria, aging, proteomics

## Abstract

Mitochondrial dysfunction is evident in numerous neurodegenerative and age-related disorders. It has also been linked to cellular ageing, however our current understanding of the mitochondrial changes that occur are unclear. Functional studies have made some progress reporting reduced respiration, dynamic structural modifications and loss of membrane potential, though there are conflicts within these findings. Proteomic analyses, together with functional studies, are required in order to profile the mitochondrial changes that occur with age and can contribute to unravelling the complexity of the ageing phenotype. The emergence of improved protein separation techniques, combined with mass spectrometry analyses has allowed the identification of age and cell-type specific mitochondrial changes in energy metabolism, antioxidants, fusion and fission machinery, chaperones, membrane proteins and biosynthesis pathways. Here, we identify and review recent data from the analyses of mitochondria from rodent brains. It is expected that knowledge gained from understanding age-related mitochondrial changes of the brain should lead to improved biomarkers of normal ageing and also age-related disease progression.

## INTRODUCTION

The mitochondrion is a ubiquitous intracellular organelle instrumental to eukaryotic existence. It is the major intracellular site of oxygen consumption and producer of the high energy molecule adenosine triphosphate (ATP). Mitochondria carry out tasks besides energy production, including cellular homeostasis and signalling, iron processing, haem and steroid synthesis, protein and lipid biosynthesis and apoptosis. These organelles are extremely dynamic and variable, capable of responding to numerous stimuli (including temperature, nutrients, hormones, exercise and hypoxia); they initiate the production of new mitochondria and their selective removal.

The brain, per gram, has the highest demand for glucose than any other tissue [1] requiring 120 – 130g glucose per day in adults [2]. Brain function is entirely dependent on glucose and oxygen from the carotid and vertebral circulation. Glucose oxidation followed by oxidative phosphorylation is accountable for the vast majority of ATP generated in the brain [3]. Brain energy metabolism declines with age [4]. Recent data from our own group [5]and others [6] have observed this decline to be clinically homogenous in most brain regions. This metabolic change is considered to be a feature of the ageing phenotype [4] as well as age-related neuro-degeneration, where there is mounting evidence supporting the role of dysfunctional mitochondria in their progression [7–10].

As our understanding of ageing has progressed mitochondrial function has come to the forefront as pivotal to the aged phenotype. Classical theories, including the mitochondrial free radical theory of ageing (MFRTA) [11], have led the field. According to the MFRTA an accumulation of oxidative damage, caused by mitochondrial free radicals, is the driving force behind ageing. However, this theory conflicts with growing evidence from animal models. Species comparison between the long lived naked mole rat and short lived mouse indicates little difference in the production of ROS between species [12] and no age-dependent variation in antioxidant enzyme expression [13]. This suggests that mitochondrial ROS may act as signalling molecules, prolonging maximum lifespan. Indeed this is consistent with data from mice [14], flies [15] and nematodes [16]. In *C. elegans*, despite a clear increase in oxidative stress due to superoxide dismutase (SOD) gene deletion, no change in lifespan was seen [17]. MFRTA also fails to fully explain the functional brain mitochondrial deficits that occur with age. These deficits include reduced respiration [18], dynamic changes in shape and size [19], activation of permeability transition pore [20] and loss of membrane potential [21]. Although functional studies have gone some way to identifying these mitochondrial changes, there is variability found in the direction and extent to which these differences occur. There is even evidence to suggest that oxidative phosphorylation activity may in fact increase with age [22–24]. There are similar inconsistencies which exist for the role and the activity of mitochondrial antioxidants [25,26], fusion and fission dynamics [19, 27] and other mitochondrial proteins with age [28]. Profiling of mitochondrial protein expression in tissues from different ages can add molecular insight, which in conjunction with functional studies can be a powerful approach towards unravelling this complexity. In this review we look at the current state of proteomic technologies and focus on evaluating findings from mitochondrial proteomic studies of brain tissue from the past decade.

### Techniques for mitochondrial proteomic analysis

Proteomics is the large scale, high-throughput identification of proteins within a biological unit – tissues, fluids or cells. First described in 1975, and still used to this day, the original proteomic technique is two-dimensional gel electrophoresis (2DE) [29]. This involves the separation of proteins initially by their isoelectric point and secondly by their molecular weight. Proteins are resolved within the biological sample into spots on a stained gel which each represent one or a small number of proteins. 2DE has well recognised limitations, reviewed by Rabilloud and Lelong (2011) [30]. 2DE analysis of membrane proteins is not suitable due to isoelectric precipitation [31]. Further, 2DE presents a limited scope of analysis of whole cell extracts [30]. However, despite the limitations it is a very economical, robust and reproducible method which has stood the test of time. It still has some utility in profiling individual cellular fractions, including mitochondria, where the sample complexity is closer to the resolving power of 2DE [7, 32].

The fluorescent younger sibling to 2DE is two-dimensional difference in-gel electrophoresis (2D-DIGE), reviewed by Diez et al (2010) [33]. 2D-DIGE involves the pre-labelling of two or more protein samples, before they are run on the same gel. They are labelled at cysteines or lysines with cyanine dye (CyDye) fluorophores (Cy2, Cy3 and Cy5). 2D-DIGE facilitates ease of comparison between two or more proteomes, reducing the number of gels, cost and time required. It includes the use of an internal pooled standard which is particularly useful for normalisation of identified spots without the need for classical pair-wise analysis. However, similar to classical 2DE it shares drawbacks in keeping hydrophobic and membrane proteins from precipitating [33]. There is also some difficulty in resolving very high or very low molecular weight or extreme isoelectric point proteins. One more gel based technique, blue native gel electrophoresis (BN-PAGE) is used for the isolation of membrane bound protein complexes BN- PAGE has been used to separate and recover the oxidative phosphorylation machinery of the inner mitochondrial membrane [34]. Mass spectrometry (MS) (reviewed by Cunningham et al, 2012) [35] is the central technology for the identification and characterisation of proteins from spots identified with 2DE/2D-DIGE/BN-PAGE. Briefly, protein spots of interest are excised, trypsin digested and masses are measured on MS-instruments to produce a peptide mass fingerprint, which is compared against an *in silico* database.

Despite the utility of 2DE/MS, 2D-DIGE/MS and BN-PAGE, and their use within mitochondrial proteomic ageing research [36–38,7], new methods have been developed with further advantages [39]. Technological advances in gel-free liquid chromatography have stepped up in delivering these. Proteomic analyses, including research within mitochondrial ageing dynamics, more frequently employ liquid chromato-graphy coupled with mass spectrometry (LC/MS-MS) [40]. Liquid chromatography comes in a variety of modes: ion-exchange, reverse phase, affinity and size exclusion chromatography (reviewed by Pitt, 2009) [41]. Each modality has its own set of positives and negatives to its use, however most proteomic separations are carried out in reverse phase - LC mode due to its compatibility with MS. LC/MS is a viable replacement for older proteomic technologies, as it provides superior specificity and sensitivity, although highly trained personnel are essential and start-up costs are large [42].

In the proteomic quantification of mammalian tissues *in vitro* and *in vivo* stable isotopic labelling (reviewed by Gevaert et al, 2008) [43] and label-free techniques are used (reviewed by Wong and Cagney, 2010) [44]. In vivo methodologies include stable isotope labelling by amino acids in cell culture and also in mammals (SILAC), and *in vitro* options include isotope-coded affinity tag (ITRAQ). These methods boast greatly increased sensitivity compared to traditional 2D-PAGE techniques, due to chromatographic separation [45]. However, as with all techniques stable isotopic labelling suffers some disadvantages in methodology. A major drawback of SILAC is its inapplicability to whole tissue protein analysis [45]. ITRAQ relies on the differential tagging of cysteine residues in proteins. However, this is a drawback due to it missing approximately 5-10% of all proteins where this amino acid is not present [45]. Further, isotope labelling requires avidin affinity and ion-exchange chromatography to be carried out, causing losses in low abundance peptides [46]. More recently, label free methods, including sequential window acquisition of all theoretical mass spectra (SWATH), have been developed to overcome the limitations of label-based quantification and its complexity of sample preparation [47]. SWATH-MS combines data independent acquisition with a targeted data extraction method. It has been demonstrated to have an equivalent standard of quantification accuracy to that of label-based methodologies [48], whilst being able to detect and quantify a significantly larger proportion of the proteome [49] and maintain reproducibility and consistency [50]. SWATH-MS has not yet seen use within the field of mitochondrial proteomics, however SWATH-MS is clearly a current leader in the field of proteomic techniques.

Proteomic methods have matured over the past few decades, however the continued progress toward increased resolution, accuracy and reproducibility will allow inclusion of lower abundance proteins and those that are less well resolved with current techniques. New methods such as SWATH-MS [47], reaction monitoring/multiple reaction monitoring [51] and SELDI-TOF-MS (surface enhanced laser desorption/ionisation of flight mass spectrometry) [52] are currently being validated and should be expected to replace older 2DE/2D-DIGE methods. Indeed within mitochondrial ageing research some of these more cutting edge technologies, including SILAC, have already been demonstrated [22,53,54].

### What does proteomics tell us about mitochondrial brain ageing?

With evidence pointing toward a pivotal role of mitochondria in neurodegenerative disease and the aged phenotype, an understanding of the changes to the proteome is warranted. Searching within the *NCBI Pubmed* database using the terms ‘proteomics and mitochondria and aging or ageing’ supplied a total of six relevant proteomic studies to this review since 2006. Further, a number of reviews have been published in the past 5 years within the mitochondrial proteomic paradigms of neurodegenerative disease [55] and muscle ageing [56], illustrating the current popular interest that exists for this subject. For this review we focus upon the mitochondrial proteomic studies within brain tissue in mice [22,38,7,53,54] and rats [37], from the last 10 years.

The six mitochondrial proteomic studies found relevant to this review (summarised in Table 1) used a variety of experimental approaches. Studies used either 2DE+MS (3 out of 6 papers) [37,38,7], or non-gel based SILAC + HPLC/MS-MS (3 out of 6 papers) [22,53,54]. All mice used were CB57L/6 or CB57L/6J, whilst rats were F344/DuCrj. An inconsistency exists between the reviewed studies here in respect of the age of tissues profiled. Study designs comprised varying start and end ages, outlined in Table 1. Briefly, age ranges included young to middle aged (5 out of 6 papers), middle to old aged (3 out of 6 papers) and young to old aged (4 out of 6 papers). Although this differential study design may influence interpretation of the proteomic differences, it has proved useful in order to highlight the variation of mitochondrial protein expression that has been measured across the lifespan. We have chosen to look more broadly at brain mitochondrial proteomic alterations, including whole brain, rat left brain, individual cell types (neural progenitor cells) and cell compartments (synapse) within this review. This overview may lead to a more complete understanding of the complex differences of how mitochondrial ageing manifests itself between brain regions and cell types.

**Table 1:** 

Year of Publication	Reference	Tissue/Cells	Samples	Age range profiled	Technique used	Major findings
2006	Mao et al.,	Whole brain	C57BL/6 mice	5 - 24 months	2DE/MS	Energy metabolism and HSP changes. Decreased protein expression of complexes I and IV and an increased expression of complex V of the electron transport chain with age. HSP10 decreased expression with age.
2007	Groebe at al.	Left brain	F344/DuCrj rats	5 - 17, 17 - 31, 5 - 31 months	2DE/MS	Energy metabolism, HSP and haemoglobin subunit changes. Decreased protein expression of complex V with age. Complex IV decreased expression at 17 - 31 and 5 - 31 months. 1 TCA cycle protein upregulated at 5 - 17 months and downregulation of two proteins at 17-31 and 5 - 31 months. HSP70 decreased expression from 5 - 31 months. Haemoglobin subunit alpha upregulated at 5 - 17 months and downregulated at 17 - 31 months.
2011	Stoll et al.	Neural Progenitor Cells	C57BL/6 mice	3 - 18 months	SILAC (labelled) LC/MS-MS	Energy metabolism, HSP, VDAC and protein/lipid biosynthesis changes. Decrease in complex V protein expression. 2 TCA cycle proteins upregulated and 3 downregulated. 1 VDAC protein downregulated. 1 HSP downregulated and 2 upregulated with age. 2 protein biosynthesis proteins upregulated. 2 lipid biosynthesis proteins upregulated and 1 downregulated.
2014	Stauch et al.	Synapses	C57BL/6 mice	5 - 12, 12 - 24, 5 - 24 months	SILAC (labelled) LC/MS-MS	Energy metabolism, antioxidant, fusion and fission protein expression changes. Decreased protein expression of complexes I, III, IV, V, 3 antioxidant proteins and 3 fusion related proteins; increased expression of 1 fission related protein at 5 - 12 months. Opposite expression profile from 12 - 24 months. Increased protein expression of complex II from 5 - 24 months.
2015	Stauch et al.	Whole brain	C57BL/6 mice	5 - 12, 12 - 24, 5 - 24 months	LC/MS-MS	Energy metabolism, antioxidant, protein and lipid biosynthesis, haemoglobin subunit, fusion and fission protein expression changes. All ETC complexes, 9 TCA cycle proteins, 7 lipid biosynthesis proteins, 5 protein biosynthesis proteins, 2 antioxidant proteins and 1 fusion related protein increased from 5 - 12 months. 2 antioxidant proteins decreased from 5 - 12 months. Opposite profile from 12 - 24 months, plus 2 fission related proteins downregulated. Few changes from 5 - 24 months. 1 VDAC protein increased from 5 - 12 months, subsequent decrease from 12 - 24 months. 1 HSP protein upregulated and 1 downregulated from 5 - 12 months. 2 HSPs downregulated from 12 - 24 months. Haemoglobin subunit beta downregulated from 5 - 12 and 5 - 24 months.
2016	Pollard et al.	Whole brain	C57BL/6 mice	4-11 and 78 weeks	2DE/MS	Energy metabolism and Carbonic Anhydrase II expression changes. Increased expression of complex I. Deacreased expression of 1 TCA cycle protein. Increased expression of carbonic anhydrase II.

### Oxidative phosphorylation

Mitochondrial oxidative phosphorylation (OXPHOS) is a process of electron transfer through complexes I, II, III and IV situated on the inner mitochondrial membrane (IMM). Electrons are released into the inter-membrane space and re-enter through the F0 subunit of complex V (ATP synthase) and are utilised to form ATP by F1-ATP synthase. Electron transfer proteins have been measured as decreased in ageing brain [57], with most prominent changes seen in complexes I, III, IV and V [58-60]. Taken together, proteomic studies have generally found similar reductions in OXPHOS protein expression within whole brain tissue. A decrease in expression of proteins associated with complex I (NADH-ubiquinone oxidoreductase 1 alpha subcomplex 5 and NADH-ubiquinone oxidoreductase 13kDa-a subunit) from 12-24 [54] months old and over the lifespan at 5 – 24 [38] months old is seen. Proteomic profiling of complex II, similar to functional study reports, does not show significant change with age in all but one whole brain profile, with an increase in succinate dehydrogenase flavoprotein subunit from 5 – 12 months old and a corresponding decrease from 12 – 24 months old [54]. Interestingly, in contrast to functional studies, within whole brain and isolated brain regions the expression of proteins associated with complex III show no change in all but one proteomic profile, in which an increase in cytochrome b-c1 complex subunit Rieske is increased from 5 – 12 months old and decreased from 12 – 24 months old [54]. In mouse whole brain complexes IV and V show a general increase in expression at 5 - 12 months old [54] and a decrease in expression from 12 – 24 months old [38] with multiple subunits of complex IV (cytochrome c oxidase subunit Vb, cytochrome c oxidase subunit Va preprotein) and complex V (ATP synthase mitochondrial F0 complex subunit F, ATP synthase subunit alpha; subunit beta; subunit delta) being affected. In rat brain however, a decrease in complex IV activity is seen at 17 – 31 months old and 5 – 31 months old, however downregulation of complex V is seen at all time points (5 – 17, 17 - 31 and 5 – 31 months old) [37]. It is clear there is an overall reduction in OXPHOS protein expression in advanced age, with major expression changes in complexes I, IV and V. These results can be interpreted a number of ways. It may be that there is a reduction in overall energy metabolism or an increased propensity for glycolytic energy production. As the central producer of ROS this modulation of expression may be in response to the increased free radical induced stress, either as a consequence of ROS signalling or oxidative damage to the protein complexes.

Looking at the RNA level, previous mitochondrial gene expression analyses have found similarities to the pattern revealed within the proteome [61]. A similar ‘time-course’ to mitochondrial protein expression, with increased gene expression of complexes I, III, IV and V in 12 and 18 month old mice compared to 2 months old and a decreased expression in 24 month olds. A mitochondrial proteomic study looking at mouse brain over the lifespan (5 – 24 months old) found limited changes in OXPHOS protein expression, however from young to middle aged and middle to old aged showed distinct expression changes [54]. In whole brain from 4-11 week and 78 week old mice an increase in expression of a single subunit of complex I (NADH dehydrogenase flavoprotein 2) was seen [7], whilst only a decrease in protein expression of complex V was apparent in neural progenitor cells (NPCs) taken from 3 and 18 month old mice [53]. Taken together, these data suggest that the proteomics of mitochondrial ageing are acutely sensitive to stage of life and a simple snapshot in time is not sufficient to deduce the role that differential mitochondrial proteomics can have over the lifespan.

Interestingly, synaptic mitochondria showed a clearly contrasting pattern of OXPHOS protein expression changes when compared with whole brain [22]. The synaptic cell region, where neuronal communication occurs, is reliant on an efficient supply of ATP for vesicle mobilisation and maintenance of membrane potential [62]. In synaptic mitochondria a decrease in protein expression of complexes I, III, IV and V at 5 – 12 months old was followed by an increase in protein expression of complexes I, III, IV and V at 12 – 24 months old. Similar to a whole brain lifespan proteomic comparison [31], within the synapse little difference was found in protein expression levels of any complex at 5 – 24 months old, with only an increase in expression of complex II seen. The brain mitochondria from whole brain and individual regions of neurones can be expected to be more heterogeneous due to the number of cell types that would be included. This look at an individual neuronal cell compartment, the synapse, highlights the differential mitochondrial protein expression levels that may occur within different cell types and regions in order to compensate for specific requirements at specialized areas within a cell.

### TCA cycle

The tricarboxylic acid (TCA) cycle takes place within the mitochondrial matrix and is tasked with the conversion of pyruvate, generated from glycolysis, into acetyl-CoA. Acetyl-CoA is further converted into CO2, resulting in the production of reducing equivalents such as NADH for the electron transport chain, and the eventual production of ATP. Few studies have looked at TCA cycle proteins specifically with age. In the flight muscle of drosophila a reduction in aconitase activity is seen with advancing age [64]. Aged mouse kidney mitochondria also show decreased expression of aconitase and α-ketoglutarate dehydrogenase, and conversely an increased expression of isocitrate dehydrogenase [65]. Within Parkinson's disease there is reduced activity of the α-ketoglutarate dehydrogenase complex [66], which may pre-dispose affected regions to damage. Dysregulation of the cycle at any step may lead to inefficiencies in OXPHOS and thereby disrupt mitochondrial bioenergetics.

Two of the studies fail to make any mention of TCA cycle proteins, either focussing specifically on other proteins or finding no changes (2 out of 6 papers) [22,38]. Otherwise, a common theme in 5 – 12 month old mouse whole brain[54] and 5 – 17 month old rat brain [37] was an increase in expression of TCA cycle proteins (isocitrate dehydrogenase, isocitrate dehydrogenase 3, oxoglutarate dehydrogenase, malate dehydrogenase, fumarate hydratase, pyruvate dehydro-genase E1 alpha 1, aconitate hydratase, dihydrolipoyl dehydrogenase, succinyl-CoA ligase). A subsequent decrease in the same proteins from 12 – 24 months old in mice [54] and 17 – 31 months old in rats [37] followed. Similarly to OXPHOS protein changes over the entire lifespan, fewer changes were seen. From 5 – 24 months old, one mouse study failed to find any changes in TCA cycle protein expression [54], whilst a whole lifespan study in rats [37] showed decreases in two TCA cycle proteins only (isocitrate dehydrogenase 3 and malate dehydrogenase). Looking at NPCs taken from 3 and 18 month old mice and 4-11 and 78 week old mice whole brain some clue is gained as to the progression of changes in mitochondrial protein expression with increasing age, and further evidence for the heterogeneity of brain tissue. In whole brain only one protein has been found to show expression changes; pyruvate dehydrogenase is decreased [7], whilst in NPCs three TCA cycle proteins are downregulated at 18 months old (oxoglutarate dehydrogenase, citrate synthase and fumarate hydratase), while two are upregulated (malate dehydrogenase and aconitate hydratase) [53]. These differential findings at various ages and in whole brain, neuronal compartments and cell types highlights the necessity for a comprehensive understanding of mitochondrial proteomic dynamics over the lifespan.

To summarise, mitochondrial proteomics reveal a decreased expression of both TCA cycle and OXPHOS proteins with advanced age. A decrease in the production of reducing equivalents from the TCA cycle attenuates the feed into the electron transport chain, for the synthesis of ATP. Decreased TCA cycle protein expression may therefore indirectly reduce age related mitochondrial ROS burden.

### Antioxidants

Evidence for the changing regulation of mitochondrial antioxidants is mixed. It is seen that the production of mitochondrial superoxide does increase with age and increased oxidative damage is seen in the elderly [67]. In muscle, mitochondrial antioxidant activity is increased to compensate for this [68], however there are also studies that suggest little change or decrease in protein and mRNA levels of these mitochondrial enzymes occurs with age [69]. Looking at the naked mole rat, the longest lived rodent species with a maximum lifespan of 25-30 years, mitochondrial ROS production is very similar to the short lived mouse, with a maximum lifespan of 2-3 years [12]. However, there are no age-related variations in antioxidant enzyme expression [13], whereas ROS damage continues to increase with age [70]. Although mitochondrial antioxidants are critical to an organisms survival, as of yet these data show no strong evidence for the necessity of modulation of mitochondrial antioxidants with advancing age.

Four of the mitochondrial proteomic studies found no changes in antioxidant levels with age (4 out of 6 papers) [37,38,53,7] - those that do mirror the uncertainty in the literature. In mouse whole brain a mix of up and downregulated antioxidants is seen at 5 – 12 months and 12 – 24 months old [54]. Superoxide dismutase 2 (SOD2) and peroxiredoxin-3 (PRDX3) are upregulated and glutathione S-transferase 1 and gamma-glutamyltransferase 7 (GGT7) are downregulated at 5 – 12 months old. At 12 – 24 months old SOD2 is down-regulated and PRDX2 and GGT7 are upregulated. Comparatively, isolated mitochondrial synapses show a clear pattern of downregulated antioxidant proteins at 5 – 12 months old and upregulation at 12 – 24 months old (catalase, PRDX5, SOD2) [22]. Both synaptic and whole brain isolated mitochondria from 5 – 24 months old show no differences in antioxidant producing protein levels over the lifespan [22,54]. Too few data currently exist from proteomic profiling to do anything but add to the confusion surrounding the understanding of the role antioxidants play in the ageing phenotype.

Much effort has been directed towards understanding antioxidants and their role in combating increased oxidative damage with progressive age, yet so far little evidence exists from functional studies to solidify their importance. Proteomic studies of the mitochondria with age have also failed to clarify their importance. Accumulating evidence from long-lived species [12,13], little evidence supporting a role in lifespan enhancement [17,71–74] and evidence supporting the role of free radicals as signalling molecules beneficial to adaptive ageing[75,76] should move us past long held perceptions. Antioxidants may not be as important as we previously thought them to be to lifespan extension or to ageing. Much more effort should be placed into understanding the role antioxidants play in the general maintenance of mitochondrial health.

### Heat shock proteins

The first line of cellular defence are proteases and chaperones, including heat shock proteins (HSPs). HSPs are just one family of proteins tasked with surveillance of cellular faults to ensure repair, degradation or removal. HSPs have many functions within the mitochondria, including the maturation of mitochondrial protein imports from the cytosol [77]. Most proteins are imported into the mitochondria in a pre-protein, unfolded state. Final destination transport and folding are in part mediated by HSPs. HSP70 and 90 deliver pre-proteins to the outer mitochondrial membrane receptor subunits of the Translocase of the Outer Membrane (TOM) complex for onwards import into the matrix [77,78]. The mitochondrial compartments each contain their own chaperone pool. Within the matrix the main chaperone is mitochondrial HSP70 (mtHSP70). The role of mtHSP70 is not fully understood, however evidence suggests it maintains the inward movement of pre-proteins from the cytosol into the mitochondria through conformational change of mtHSP70 together with ATP hydrolysis [79,80]. MtHSP70 is involved in assisting protein folding in the matrix [81,82] and it has been shown that it may be a pro-apoptotic factor [83]. After release of the substrate by mtHSP70, HSP60/HSP10 chaperonin system interacts with the incompletely folded protein [84,85]. The HSP60/HSP10 complex is critical for proper protein folding as loss of function leads to an accumulation of protein aggregates [85]. Similarly to mtHSP70, there is some evidence to suggest that HSP60/HSP10 are pro-apoptotic [86]. HSPs are clearly extremely important proteins for maintenance of mitochondria. Any modulation of their expression may therefore have a critical impact upon mitochondrial function.

Increased protein misfolding is a hallmark of ageing and neurodegenerative disease where mitochondrial HSPs have been found to change expression. Import efficiency of mitochondrial proteins involved in repair of oxidatively damaged mtDNA has been seen to decrease with age [87,88]. A histological hallmark of certain neurodegenerative diseases, including Parkinson's disease and Lewy body dementia is the accumulation of fibrillary aggregates known as Lewy bodies (LBs) [89]. HSP90 and HSP60 are found extensively as components of LBs [90–93]. The accumulation of these aggregates is primarily due to protein misfolding [94]. In Parkinson's disease LBs are found within the cytoplasm of dopaminergic neurones of the substantia nigra [94]. A loss of dopaminergic neurones of the substantia nigra and thus its dopaminergic projections to the striatum mark the pathology of Parkinson's disease [94]. A recent proteomic study in mouse substantia nigra and striatum over the lifespan showed region specific differences in HSP expression [95]. The group found a loss of HSP60 in the substantia nigra only. No change in expression of HSP90 was found in either the striatum or substantia nigra. A decrease in expression of HSP60 within the substantia nigra with age could promote an environment more suitable to the accumulation of LBs in Parkinson's disease. Ageing has also been seen to cause a reduction in HSP90 expression in response to thermal stress in mesenchymal stem cells [96], liver cells [97] and lymphocytes [98]. Further, aged animal tissues and elderly human blood have shown a reduced production of HSP90 following thermal stress [99]. The tissue heterogeneity of mitochondrial HSP expression with age has also been addressed recently. A tissue specific change in mtHSP70 was shown, with increased expression in 24 month old rat kidney and lungs, decreased expression from 18 – 24 months old in testis and no change in liver with ageing [100].

Four proteomic studies found changes to the expression of HSPs with age [37,38,53,54]. In mouse brain, HSP60 was found to increase its expression from 5 – 12 months old and subsequently decrease at 12 – 24months old[54]. The partner to HSP60, HSP10, was also found to decrease with advanced age in two mouse brain models, from 12 – 24 months [54] and 5 – 24 months old[38]. mtHSP70 was found to decrease its expression in two mitochondrial proteomic profiles, in NPCs from 3 – 18 months old [53] and in rat brain from 17 – 31 months old [37]. Transporter of pre-proteins from the cytosol to the mitochondrion, HSP90, was also profiled by two papers [53,54]. In NPCs there was an upregulation in HSP90ab1 and HSP90b1 from 3 – 18 months old [53], whereas in mouse brain from 5 – 12 months old it was seen to decrease [54].

What is clear from functional studies is that HSPs remain an unknown quantity in regards to expression changes with age, and mitochondrial HSPs even more so. HSPs are involved in protein quality control, trafficking of proteins into the mitochondria and correct folding of incoming proteins. As an increased aggregation of misfolded proteins is a known occurrence with age it would be logical to assume HSPs would be upregulated. Indeed, within functional studies there is evidence to suggest tissue specific increases in mitochondrial HSPs with ageing [100]. However, proteomic data do not indicate that this is the case within brain tissue in advanced age. Brain region specific differences could be a reason for this, or there may have been a rise in HSP levels with progressive ageing, however HSP defences may eventually fail in advanced age due to overwhelming proteotoxic stress. Recently, mitochondrial HSPs have been proposed to also be pro-apoptotic factors [83,86]. There is also a decrease in global apoptosis with progressive age [101]. Therefore, this decrease in brain mitochondrial HSPs could be considered to play a role, as a cause or a consequence, of reduced apoptosis. Both functional and proteomic studies looking into this are warranted. The complexity and limited current knowledge of mitochondrial HSP expression is apparent. It is likely the degree of expression of these proteins is varied with oxidative stress, tissue, region and cell type, disease progression and likely a multitude of other variables. However, with key roles in mitochondrial maintenance and protein folding and evidence indicating expression changes with age and within regions involved in neurodegenerative disorders, they are a family of proteins of immense therapeutic potential.

### Fusion and fission

Fusion and fission are counters of each other. Fusion and fission dynamics are strongly influenced by energy and supply balance. Nutrient-rich environments are associated with a fragmented mitochondrial network, whereas fasting is associated with mitochondrial elongation [102–104]. The morphology of mitochondria have a critical impact on ATP production, with elongated mitochondria being essential to maintain ATP production and cellular survival, whereas mitochondrial fragmentation decreases ATP production and increases mitochondrial uncoupling and nutrient storage [102,103]. Mitochondrial fission may also cause mitochondrial depolarisation leading to whole organelle turnover, known as mitochondrial autophagy or mitophagy [105].

Alterations of mitochondrial dynamics can cause mitochondrial dysfunction and cellular senescence [106]. In Alzheimer's disease there is an increase in mitochondrial fragmentation coupled with increased autophagy [107–109]. Parkinson's disease patients show an increase in mitochondrial fragmentation due to increased fission protein Dynamin-1-like protein (DRP1) and cleavage of fusion protein Optic atrophy 1 (OPA1) [110]. In Purkinje cell degeneration (*pcd*) mice, a model of neurodegenerative disease, degeneration of Purkinje cell neurones is thought to be caused by over-activation of the macroautophagy pathway and mitophagy, indicating that an altered autophagy pathway may be common within neurodegenerative diseases [111]. Ageing seems to accompany a shift toward more fusion events, resulting in elongated, enlarged mitochondria[28]. It has recently been shown that a loss of DRP1 is found during senescence in aged mice endothelial cells, causing an upregulation of ROS [112]. Recent studies in mouse hypothalamic neurones have implicated a lack of Mitofusins (Mfn1 and Mfn2) with whole-body energy homeostasis regulation [113–115]. Mfn2 deficiency was found to reduce endoplasmic reticulum (ER) communication and prevent changes in mitochondrial dynamics, causing insulin resistance and hypothalamic leptin resistance; thereby leading to obesity [116,117]. Tight contact between the ER and mitochondria is pivotal for mitochondrial and ER health, as the ER provides important resources, including calcium ions and mitochondrial lipids. ER stress induced by Mfn2 deficiency is known to promote the pro-inflammatory transcription factor NF-kB [118]. NF-kB family of transcription factors is capable of stimulation by a variety of inflammatory, oxidative and genotoxic stresses; therefore, it is very likely to have involvement within ageing [119]. In the hypothalamus of mice, inhibition of NF-kB signalling in microglia was capable of extending lifespan [120]. This suggests that increased Mfn2, and thus increased fusion, may provide benefits to ageing and improved lifespan through a reduction of NF-kB. Age-related decrease in mitochondrial tethering to the ER, including ER stress-induced reduction in insulin signalling, may be an important age-associated regulatory change. Although correlation exists between mitochondrial elongation and ageing, the exact contribution is still debated. It has been shown that prolonged elongation of mitochondria results in greater intracellular ROS production and lower mitochondrial respiration rate [121]. On the other hand, in response to cellular stress, mitochondrial fusion allows mito-chondria to possess more cristae, maintain ATP production, prevent mitochondrial membrane depolarisation, inhibit cytochrome C release and to escape from mitophagy and apoptosis [122]. Therefore, mitochondrial fusion may be important in times of cellular stress to allow mitochondria to regain cellular functionality.

Only two mitochondrial proteomic studies have found alterations in fusion and fission mechanics regulation [22,54]. Both are in agreement that a reduction in fission is seen from 12 – 24 months old (DRP1 and mitochondrial fission factor) in mice [22,54]. An increase in fission from 5 – 12 months old is also seen within synaptic mitochondria only [22]. Fusion mechanics are less well defined with age from the proteomic studies that have characterised them. Within a whole brain model, from 5-12 months old fusion increases (MFN1), whilst from 12 – 24 months old it is seen to downregulate (OPA3, MFN1) [54]. Within synaptic mitochondria however, the opposite was found: fusion proteins are downregulated from 5 – 12 months old (MFN1, MFN2, OPA1) and upregulated from 12 – 24 months old (MFN1, MFN2, OPA1) [22]. Due to the high reliance of the synapse on an efficient supply of ATP, the upregulation of fusion proteins with advancing age may be a compensatory mechanism to rescue cellular functionality through maintaining ATP production, increase number of cristae, prevent mitochondrial membrane depolarisation, inhibit cytochrome C release and escape from mitophagy and apoptosis.

### VDAC

Voltage dependent anion channels (VDAC) are the most abundant protein in the outer mitochondrial membrane (OMM). They are an important component of the mitochondrial permeability transition pore, facilitating the trafficking of <5kDa metabolites through the OMM and are involved in ER – mitochondria crosstalk [123]. It has been shown that superoxide produced by complexes I [124] and III [125] are released from the mitochondria through VDACs in times of pathological stress or decreased mitochondrial antioxidant levels. VDAC is thought to participate in the intrinsic apoptosis pathway. The intrinsic apoptosis pathway involves the permeablisation of the OMM, as a mitochondrial transition pore. VDAC is involved in cytochrome C release, although the exact mechanism is under dispute [126]. VDACs come into direct contact with both pro- and anti-apoptotic factors [127,128]. The complexes formed through VDAC/apoptotic factor binding are poorly understood although their structures have been predicted [129]. The precise role for VDAC within the apoptotic cascade is currently unknown.

The dysregulation of mitochondria-mediated apoptosis has a role in ageing [130] and neurodegenerative disease [131–134]. However, the direction of apoptotic protein expression, enhancement [135] or suppression [101], is questioned. On the one hand too little apoptosis can lead to accumulation of dysfunctional cells, while too much can cause degeneration of tissues. Recent evidence in humans has pointed toward a decrease in apoptosis with age, including a decrease in pro-apoptotic proteins and apoptotic markers and an increase in anti-apoptotic proteins [101]. As VDAC is a component of apoptosis through its interactions with apoptotic proteins and is involved in cytochrome C release its expression in ageing and neurodegeneration may be expected to change. Indeed, in Alzheimer's disease there is an increase of VDAC2 in the temporal cortex and a decrease of VDAC1 in frontal cortex [136]. In familial ALS, post-translational modifications of VDAC2 have been reported [137]. Further, in the hippocampus of an epileptic model mouse, a 3-fold increase in VDAC1 and 4-fold decrease in VDAC2 was seen [138]. A study on VDAC expression with age within a human ageing model, human umbilical vein endothelial cells (HUVEC), and aged *Drosophila* found a change in expression and post-translational modifications [139]. In *Drosophila* a decrease in expression was found in 3 isoforms of VDAC1 and an increase in expression was found in one. In HUVEC an increase in expression was found in VDAC2. Currently two mitochondrial proteomic papers have profiled VDAC proteins with age [53,54]. Here there is agreement that the expression of VDAC1 decreases in advanced age [53,54]. No other VDAC family member showed expression change with age. There was also found to be an increase in VDAC1 from 5 – 12 months old [54], once again hinting at a time-course to mitochondrial protein expression within the VDAC family of proteins as well. As VDAC is suggested to be involved in the apoptotic cascade through multiple mechanisms, including cytochrome C release and interaction with apoptotic proteins, the decrease in VDAC protein expression shown from these proteomic analysis with advanced age is in agreement with decreased apoptosis in normal ageing.

### Haemoglobin

Haemoglobin (Hb) is well known as a transporter of oxygen, carbon dioxide and nitric oxide, however it is also involved in regulation of gene expression, nitric oxide signalling and terminal oxidase activity [140]. It is a heterotrimeric protein comprised of two alpha (Hba) and two beta (Hbb) subunits. Within each subunit of Hb is a haem group with an Fe2+ core which allows Hb to bind gaseous molecules. A recent finding is the localisation of Hba and Hbb to the mitochondrial inner membrane [32]. It has been postulated that a close proximity to the oxidative phosphorylation machinery may be important for maintenance and support in times of oxidative stress.

Hba and Hbb are expressed within neurones and localisation to the mitochondria is known to occur within the substantia nigra and frontal lobe [32]. Hba and Hbb mRNA are strongly downregulated within the brain with age [141] and within animal models of neurodegeneration[142]. Post-mortem PD patient substantia nigra [32,143] and medulla oblongata [143] have reduced Hba and Hbb. It has been shown that mitochondrial Hba and Hbb are decreased in 2 – 18 month old control and neurodegenerative model mice [32]. Two proteomic papers have so far described the protein expression of Hba and Hbb within ageing brain mitochondria [37,54]. Similarly to functional studies within the brain Hba and Hbb have been found to decrease with ageing. In mice Hbb protein expression decreased from 5 – 24 months old [54] and in rats Hba expression decreased from 17 – 31 months old [37]. From young to middle aged Hba and Hbb have varied expression. Hba increases from 5 – 17 months old in rats [37] and decreases from 5 – 12 months old in mice [54]. There are currently limited mitochondrial papers profiling the subunits of Hb - perhaps in some cases this is due to relegating it to cytosolic status and subsequent removal from the published data. With decreased expression changes of Hba and Hbb in functional studies of ageing [141] and neurodegeneration [104,106,107], and agreement from mitochondrial proteomic profiling, it would be prudent for further studies to elucidate whether Hb expression changes are a cause or consequence of the process.

### Carbonic anhydrase II

Carbonic anhydrases (CA) are a family of zinc metalloenzymes tasked with catalysis of the reversible hydration of carbon dioxide (CO_2_) to bicarbonate (HCO_3_^−^). Further they are involved in the exchange of carbon dioxide in tissues and the formation of bodily fluids, including aqueous humour, saliva, bile, urine, pancreatic juice, gastric secretion, sweat and cerebrospinal fluid [144]. In mammals there is the α family, consisting of at least 16 different CA isoforms, which differ by tissue location, subcellular localisation, catalytic activity (reviewed by Supuran, 2016) [144]. For such a ubiquitous family with wide ranging tasks there is a lack of knowledge as to the pathophysiological effect their dysfunction may have, not least within ageing.

Carbonic anhydrase II (CAII) is a widely distributed isoform – present in the cytosol of a large number of cell and tissue types. It is the target of inhibitors for the treatment of glaucoma[145], cancer [145] and epilepsy [146]. Further, the CAII inhibitor methazolamide has recently been shown to be neuroprotective in a mouse model of Alzheimer's disease [147]. Recent mito-chondrial proteomic profiles of mouse brain at 5 – 12 months old [54] and 4-11 to 78 weeks old [7] have shown an increase in CAII levels. Increased CAII levels with age have also been identified in various other tissues, including mouse retina [7], rat muscle [148,149], kidney [150] and human muscle [151]. These data suggest CAII associates with strong affinity to the mitochondrial outer membrane. Recent work by our group has made some progress in understanding the role of CAII in ageing [7]. In the long-lived nematode, *C.elegans*, a reduction in lifespan was found with the addition of CAII. Increased CAII expression is not exclusively an ageing phenomenon. In the *pcd* neurodegenerative mouse model a significant increase in CAII was seen in brain cerebellum and retina [7]. Evidence points towards upregulation of CAII as an event of both ageing and neurodegeneration. It is likely that upregulation of CAII is a feature of other neurodegenerative diseases, not limited to glaucoma [145]. Increased expression of CAII from ageing brain mitochondrial proteomic profiles and lifespan reduction of *C.elegans* by addition of CAII clearly displays it as an exciting novel regulator of ageing that warrants further study to elicit its role in this process. The possibility of CAII upregulation in neurodegenerative diseases also requires investigation as currently in use carbonic anhydrase inhibitors may have wider application.

### An overview

Recent mitochondrial proteomic analyses of brain tissue from mice and rats has provided many more questions than answers to the already conflicting results from functional studies. It is clear that oxidative phosphoryla-tion complexes and TCA cycle proteins are affected by age, however the directionality and extent to these changes is in question. Proteomic analyses of whole brain, neuronal compartments and specific cell types have highlighted the heterogeneity of mitochondrial protein expression changes with age. Looking at whole brain samples, the consensus from these proteomic studies is a decrease in expression of TCA cycle proteins and complexes I, IV and V, with some evidence for decreases in complexes II and III expression. Synaptic datasets suggest an opposing proteomic profile. Future work in this area will need to ensure an understanding of this potential for differential mitochondrial protein expression between regions and cell types.

Age ranges used for profiling in these studies cover the lifespan, highlighting an important point that similar to findings from gene expression analyses, a dynamic time course for mitochondrial protein expression exists [61]. In whole brain OXPHOS complexes and TCA cycle this tends to be an upregulation from young to adult and a corresponding downregulation from adult to old. Whole lifespan changes seem to be minimal, perhaps reflecting a see-saw effect of protein expression changes with age. Special consideration should be taken when planning future experimental work in this area to consider these changes at different stages of life. Further work should focus upon elucidating the mitochondrial proteomic changes at smaller time intervals in order to define 'age boundaries' for future ageing-targeted therapies.

A previously limited area of knowledge was mitochondrial HSP expression with age. This review has brought together the evidence, from proteomic profiling, that mitochondrial specific HSPs (HSP10, 60, 70) are downregulated in old aged whole brain tissue. Previous age related functional and proteomic studies have found tissue specific expression patterns, with increased mtHSP70 in rat kidney and lungs, decrease in rat testis and no change in liver with ageing [100]. Another group found brain region specific differences in HSP60 levels with a loss in the substantia nigra and no change in the striatum [95]. Due to this loss in protein folding capability, age may create an environment more suitable for substantia nigra mitochondrial dysfunction, and thus the onset of Parkinson's disease. Since there is agreement from the mitochondrial proteomic profiles that an overall reduction in mitochondrial HSP expression with old age exists, further study should address expression changes within specific brain regions and the impact decreased expression, and its alteration, may have on lifespan and neurodegenerative disease.

Taken together, in whole brain there seems to be a mitochondrial proteomic movement toward decreased apoptosis with advanced age. Downregulation of OXPHOS complexes has been shown to reduce susceptibility to apoptosis [152]. Potentially pro-apoptotic mitochondrial HSPs [83, 86] and VDACs [126] are seen to have decreased expression in the mitochondrial proteomic profiling here. Mitochondrial fission is an early event in apoptosis [153] and is a mediator of cytochrome C release [154]. With the decreased expression of mitochondrial fission proteins here, this would suggest an anti-apoptotic phenotype. An overarching question still posed is the direction of apoptosis with ageing. Analysis of mitochondrial proteomic profiles would suggest an anti-apoptotic phenotype in whole brain.

Brain mitochondrial proteomic profiling has pulled out intriguing novel proteins modulated by age. Carbonic anhydrase II is one such protein, suggested to have strong affinity to the mitochondrial outer membrane. Two brain mitochondrial proteomic studies found an increase in CAII expression in young to adult mice [7, 54], indicating a role in the regulation of ageing. Addition of CAII in *C.elegans* has corroborated its importance by producing a decrease in lifespan [7]. CAII holds exciting possibilities as a novel regulator of ageing, however little understanding of its role is currently known. With CAII inhibitors in use for glaucoma [145], cancer [145] and epilepsy [146], an exciting possibility is its use to improve ageing outcomes.

As proteomic technologies have improved a number of the more recent proteomic studies within this review have moved away from gel-based techniques. Using newer technologies a greater number of mitochondrial protein expression changes were seen. SILAC combined with LC/MS-MS boasts improved sensitivity for measurement of lower abundance proteins [40], however the cost and its inability to directly analyse tissues are drawbacks to its use [45]. The use of the older methodology 2DE still works for these mitochondrial proteomic profiles finding fewer protein expression differences, however the small size of the mitochondrial proteome makes it suitable for this use. Ageing and mitochondrial antioxidants, VDAC proteins, fusion and fission protein dynamics, haemoglobin subunits, heat shock proteins and carbonic anhydrases have varied, or limited, description in functional studies. As future technologies, including SWATH-MS [47], with the ability to measure at a greater resolution, reproducibility and higher sensitivity become more widespread we may be able to profile the lower abundance proteins involved in other mitochondrial functions: biogenesis [155], mitophagy [156] and steroid synthesis [157], which have been seen to have altered expression in functional studies. Overall mitochondrial proteomic research is already challenging our ideas of ageing and can be seen to be of great utility in the future of ageing research.

### Conclusion

Mitochondrial proteomic alteration in brain ageing is clear, however the directionality and extent of these alterations is not. The application of quantitative proteomics to mitochondria is timely to more comprehensively investigate these changes. Mito-chondrial proteomics of brain ageing has elucidated expression changes in key mitochondrial proteins over the lifespan and highlighted the heterogeneity that may occur between cell types and cell regions. The establishment of biological markers of ageing will allow for a more coherent theory of brain ageing, the development of diagnostic criteria to differentiate between ageing and age-related diseases and the validation of potential targets for the improvement and elongation of health and lifespan. With the advent of new proteomic technologies, bringing greater reproducibility and accuracy, the field of mitochondrial proteomics is open-ended and improved clarity of the mitochondrial changes that occur in the brain with age is expected in the near future.
